# 107. Impact of Penicillin Allergy Assessment During Pre-Anesthesia Testing (PAT) on Beta-Lactam Surgical Prophylaxis in Bariatric Surgery Patients

**DOI:** 10.1093/ofid/ofab466.309

**Published:** 2021-12-04

**Authors:** Maggie Hitchins, Amber M Watts, Shannon Holt

**Affiliations:** WakeMed Health & Hospitals, Raleigh, North Carolina

## Abstract

**Background:**

Due to utilization of alternative antibiotics, documented penicillin (PCN) allergies are associated with an increased risk of surgical site infections, cost, and infections caused by resistant organisms. In October 2019, a community hospital implemented a beta-lactam (BL) allergy assessment service in a pre-anesthesia testing (PAT) clinic without access to allergy specialists or PCN skin testing (PST). In phase 1, the surgeon was contacted to change surgical prophylaxis for BL eligible patients based on the assessment. In phase 2, an automatic protocol was implemented to allow advanced practice providers (APPs) to switch from alternative antibiotics in BL eligible patients. The objective of this study was to assess the impact of the PCN assessment service and protocol on BL surgical prophylaxis.

**Methods:**

This retrospective cohort study included bariatric surgery patients who visited PAT clinic with a documented BL allergy between Jun 2019-Sept 2019 (control), Nov 2019-Feb 2020 (phase 1), and Nov 2020-Feb 2021 (phase 2). Patients with procedures not requiring surgical prophylaxis were excluded. Patients were determined to be eligible for BL surgical prophylaxis if: intolerance or mild-moderate reaction to PCN, previously tolerated cephalosporin, intolerance to cephalosporin, or surgeon deemed it appropriate. The primary outcome was overall utilization of BL surgical prophylaxis.

**Results:**

This study included 38 patients in the control group, 14 in the phase 1 group, and 17 in the phase 2 group. Overall utilization of BL surgical prophylaxis significantly increased with 16% in the control group, 43% in the phase 1 group, and 65% in the phase 2 group (p=0.001). In the BL eligible patient subgroup, BL surgical prophylaxis significantly increased with 35% (n=6/17) in the control group, 50% (n=6/12) in the phase 1 group, and 92% (n=11/12) in the phase 2 group (p= 0.001). There were no reported surgical site infections or adverse drug reactions.

**Conclusion:**

Overall utilization of BL surgical prophylaxis significantly increased after implementation of a PCN allergy assessment service with an automatic protocol for patients determined as BL eligible. This service and protocol demonstrates successful optimization of surgical prophylaxis when allergy specialists or PST is not available.

**Disclosures:**

**All Authors**: No reported disclosures

